# DNA as a Model for Probing Polymer Entanglements: Circular Polymers and Non-Classical Dynamics

**DOI:** 10.3390/polym8090336

**Published:** 2016-09-07

**Authors:** Kathryn Regan, Shea Ricketts, Rae M. Robertson-Anderson

**Affiliations:** Department of Physics and Biophysics, University of San Diego, San Diego, CA 92110, USA; keregan@sandiego.edu (K.R.); shearicketts@sandiego.edu (S.R.)

**Keywords:** DNA, entangled polymers, circular polymers, ring polymers, nonlinear rheology, microrheology, particle-tracking, reptation

## Abstract

Double-stranded DNA offers a robust platform for investigating fundamental questions regarding the dynamics of entangled polymer solutions. The exceptional monodispersity and multiple naturally occurring topologies of DNA, as well as a wide range of tunable lengths and concentrations that encompass the entanglement regime, enable direct testing of molecular-level entanglement theories and corresponding scaling laws. DNA is also amenable to a wide range of techniques from passive to nonlinear measurements and from single-molecule to bulk macroscopic experiments. Over the past two decades, researchers have developed methods to directly visualize and manipulate single entangled DNA molecules in steady-state and stressed conditions using fluorescence microscopy, particle tracking and optical tweezers. Developments in microfluidics, microrheology and bulk rheology have also enabled characterization of the viscoelastic response of entangled DNA from molecular levels to macroscopic scales and over timescales that span from linear to nonlinear regimes. Experiments using DNA have uniquely elucidated the debated entanglement properties of circular polymers and blends of linear and circular polymers. Experiments have also revealed important lengthscale and timescale dependent entanglement dynamics not predicted by classical tube models, both validating and refuting new proposed extensions and alternatives to tube theory and motivating further theoretical work to describe the rich dynamics exhibited in entangled polymer systems.

## 1. Introduction

Double-stranded DNA, the genetic code for nearly all life, naturally occurs in a wide range of lengths (>10^6^ orders of magnitude) and in multiple topologies, including supercoiled, relaxed circular and linear forms. Genomic DNA, condensed in the cell nucleus and packaged into viral capsids, exists in highly-entangled states; and viral DNA and gene therapy vectors must traverse the crowded cellular environment containing high concentrations of biopolymers. Beyond the biological significance of understanding DNA dynamics in entangled and concentrated states, over the past several decades, DNA has also been shown to be an excellent model system for investigating fundamental questions in polymer science [1,2,3,4,5,6,7]. This review discusses: (i) the advantages of DNA as a platform for probing polymer entanglements; (ii) the techniques used to probe entangled DNA dynamics; and (iii) the important results characterizing entangled DNA. DNA has been used extensively to investigate dynamics of single polymers and polymers in dilute phase, and a large pool of literature exists on the matter [3,4,5,8,9,10,11,12,13,14,15]. This review instead focuses on entangled and concentrated DNA, highlighting work on circular DNA and nonlinear dynamics.

The dynamics of entangled polymers can be described by the Nobel-prize winning reptation tube model, pioneered by De Gennes and advanced by Doi and Edwards, which postulates that each entangled polymer is confined to a tube-like region (formed by the surrounding entangling polymers) that restricts motion transverse to its contour [16,17]. Thus, each molecule can only diffuse “head first” in a direction parallel to its contour—a process termed reptation. The power of this mean-field theory lies in its simplicity, reducing all of the surrounding entangling polymers to a square-well confining field (or tube) with a single static tube radius *a* = (24/5(*L*_e_/*L*)*R*_G_^2^)^1/2^ where *R*_G_ is the radius of gyration, *L* is molecular length, and *L*_e_ is the molecular length between entanglements [17]. When an entangled “test” polymer is strained, the surrounding confining tube deforms but maintains its size, so the spacing between entanglements remains fixed. The polymer then relaxes this induced stress by diffusing out of the deformed tube into a new unperturbed tube, which occurs over the predicted disengagement time *τ*_D_. While complete relaxation can only occur after disengagement from the original tube, elastic relaxation of stretched polymer segments, i.e., Rouse relaxation, is also predicted to contribute to relaxation over shorter timescales *τ*_R_. The tube model framework predicts dynamical scaling laws that relate intrinsic polymer properties such as length *L* and concentration *c* to dynamical quantities such as diffusion coefficients *D*, viscosities *η*, and relaxation timescales *τ*. While the Doi–Edwards (DE) tube model has been widely successful in accurately modeling entanglement dynamics for linear polymers in steady-state and linear (small stress/strain) regimes, its application to ring (circular) polymers and to nonlinear regimes (arising from large applied strains) remains challenged.

The extension of tube theory to entangled ring polymers is complicated by the fact that rings have no ends, thereby preventing conventional reptation; and past experimental and theoretical studies lack consensus on the dynamics of entangled ring polymers. However, circular polymers are essential to fundamental life processes and emerging biotechnologies such as single cell DNA sequencing; and desired material properties including miscibility, thermal stability and strain hardening have been shown to greatly increase upon linear-to-ring conversion of polymers [18,19,20,21]. As such, the molecular dynamics and mechanical properties of ring polymers remain fervently debated [21,22,23,24,25,26,27,28,29,30,31,32,33,34,35,36,37,38,39,40,41,42,43,44,45,46]. Several theoretical extensions to the tube model have been proposed that model entangled rings as amoeba-like lattice-animals (with several loops branching out in different directions) diffusing through fixed obstacles [26,46,47,48]. These models typically borrow theoretical concepts from models originally developed for branched polymers, such as the pom–pom model [49,50,51]. However, recent DNA tracking experiments have called into question the notion of fixed obstacles for entangled rings and suggest the importance of other diffusive mechanisms [36]. Conflicting experimental results on bulk properties of entangled ring polymers [26,48,52,53,54,55,56] can be attributed, in part, to the near impossibility of achieving pure samples of synthetic ring polymers [26,42,45,57]. Ring samples typically contain varying fractions of linear polymers, as well as knotted and concatenated structures, due to the cyclization process used to convert linear polymers to rings. Moreover, rheological studies have found that the addition of even a small amount of linear polymers to entangled rings dramatically alters mechanical properties [26,43,44,45,53,54,55]. In blends of ring and linear polymers, rings can become threaded by their linear counterparts, in which case the only way they can move is by the threading linear polymers releasing their constraints (i.e., diffusively unthreading themselves). This constraint release (CR) process of diffusion is much slower than reptation, and leads to highly complex and varying dynamics.

CR is also expected to play an important role, competitive with reptation, in the nonlinear response of entangled polymers. When subject to small (linear) strains or deformations entangled polymers typically display viscous response at low frequencies (terminal regime) and principally elastic response at high frequencies (entanglement regime). In the low frequency terminal regime the storage and loss moduli (*G′*, *G″*) scale with frequency *ω* as *G′*~*ω*^2^ and *G″*~*ω* and the loss tangent *δ* = *G″*/*G′* is >1. Conversely, the entanglement regime is characterized by a frequency-independent *G′* plateau (i.e., the plateau modulus *G_N_^0^*), *G″* scaling transitioning from *ω*^−1/4^ to *ω*^1/2^, and *δ* < 1. The frequency at which *δ* = 1, termed the crossover frequency *ω*_c_, can be used to estimate the disengagement time *τ*_D_. In the high-frequency regime entangled polymers typically exhibit frequency-dependent viscosity (or shear) thinning in which the viscosity scales as *η*~*ω*^−1^ compared to η~*ω*^−0.5^ for weakly entangled or semi-dilute systems. Of note here is that the original DE tube model, which does not account for any constraint release, predicts *ω*^−1.5^ while the scaling exhibited in experiments aligns with models that incorporate CR of surrounding chains into the reptation dynamics of each test chain. The DE model further predicts *G″*~*ω*^−1/2^ in the entanglement regime rather than the *ω*^−1/4^ scaling that experiments display, with the correct scaling only emerging in theories that incorporate contour length fluctuations (CLF), in which the relatively free ends of a confined polymer are able to quickly relax with minimal tube constraints [58]. CLF and convective constraint release (CCR), in which entangled polymers convect past each other more easily than reptating to relax stress—as well as other non-reptative dynamics, such as entanglement tube dilation, shrinking and stretching—become increasingly important in the nonlinear regime in which polymers are deformed far from equilibrium (i.e., fast/large flows) [59,60,61,62,63]. Theoretical developments of these nonlinear features, aimed at resolving discrepancies between entangled polymer experiments and DE tube model predictions, have only recently been established and many of the predictions remain untested [63,64,65,66,67,68,69,70,71,72]. Further, non-classical concepts, such as tube dilation, have only very recently been extended to ring polymers [46].

## 2. Why DNA?

Double-stranded DNA is a semiflexible polymer with a diameter of ~2 nm, a persistence length of *L*_p_ ≅ 50 nm, and a corresponding Kuhn length, which defines the monomer size in freely-jointed chain polymer models, of *L*_K_ ≅ 100 nm [10,73]. Due to its unique thinness as compared to synthetic polymers, substantial entanglements can form in DNA solutions with modest concentrations (<1 mg/mL, <1% volume fraction), while the same level of entanglement with synthetic polymers requires extremely concentrated solutions or melts (with no solvent). High concentrations (~10–100 mg/mL) are also feasible without inducing nematic ordering, as seen with stiffer biopolymers such as actin and microtubules. Thus, using DNA uniquely enables systematic investigations of entanglement dynamics over several decades of concentration to directly test theoretical predictions and to motivate new theories that directly incorporate effects of concentration. We note, however, at the present moment DNA cannot be used to directly probe entangled polymer melt dynamics because DNA molecules in the absence of aqueous solution assume compact crystal structures.

While entanglement theories have largely been developed for monodisperse polymer systems, DNA is one of the few polymer platforms capable of achieving true monodispersity. Unlike samples of most synthetic polymers and polymerizing proteins, such as actin and microtubules, which have a distribution of lengths (quantified by the polydispersity index), concentrated monodisperse DNA samples of exactly the same length can be achieved simply by replication of expressed DNA in *Escherichia Coli* cells. Further, systematic cloning and PCR allow for a wide size range of DNA constructs and precision control of contour lengths (Table 1). Many of the previous entangled DNA studies have used commercially available linear lambda DNA (New England Biolabs, Thermofisher) which has a contour length of 48.5 kilobasepairs (kbp) (*L* ≅ 16 μm, *N* = *L*/*L_K_* ≅ 160). Several studies have also used 42.2 kbp circular Charomid DNA (*L* ≅ 14 μm, *N* ≅ 140; Wako, Nippon Gene) and 168 kbp T4 DNA (*L* ≅ 56 μm, *N* ≅ 560; no longer available). While calf thymus DNA (~13 kbp (Thermofisher) or ~75 kbp (USB Corp.)) has also been used, these commercially available samples are polydisperse so results cannot be directly compared to monodisperse results and theoretical predictions. D.E. Smith′s group at UCSD developed a set of DNA constructs with logarithmically-spaced lengths spanning two orders of magnitude (~3–300 kbp, ~1–100 μm) that have also been used and are available to researchers upon request (Table 1) [74]. Commercially available DNA constructs are typically only available in small volumes and at relatively low concentrations, making bulk rheology measurements of highly entangled DNA costly. However, the labs of D.E. Smith and R.M. Robertson-Anderson have developed relatively inexpensive methods to produce large quantities of highly concentrated DNA samples for the express purpose of facilitating bulk rheology studies of entangled DNA [32,74].

Expressed bacterial DNA (most commonly used in molecular biology and polymer studies) is typically supercoiled; however, both linear and ring (relaxed circular) topologies can be easily produced by enzymatic conversion of supercoiled constructs [5,74]. Further, the ability to reliably produce stable ring constructs, via enzymatic relaxation of supercoils in unknotted circular DNA using Topoisomerase I, is largely unique to DNA as cyclization processes used for synthetic polymers typically involve annealing the ends of linear polymers which can introduce knotting, concatenation and linear contaminants [18,19,20,75]. Great strides have been made in recent years to improve the cyclization of synthetic polymers by means of fractionation by liquid chromatography at the critical condition (LLLC), increasing purity to >99% [41,42,45]. Nonetheless, knotting and concatenation are still unavoidable and experiments using these state-of-the-art techniques still find signatures of linear and concatenated contaminants that alter the dynamics of entangled rings [42,45].

**Figure 1:**
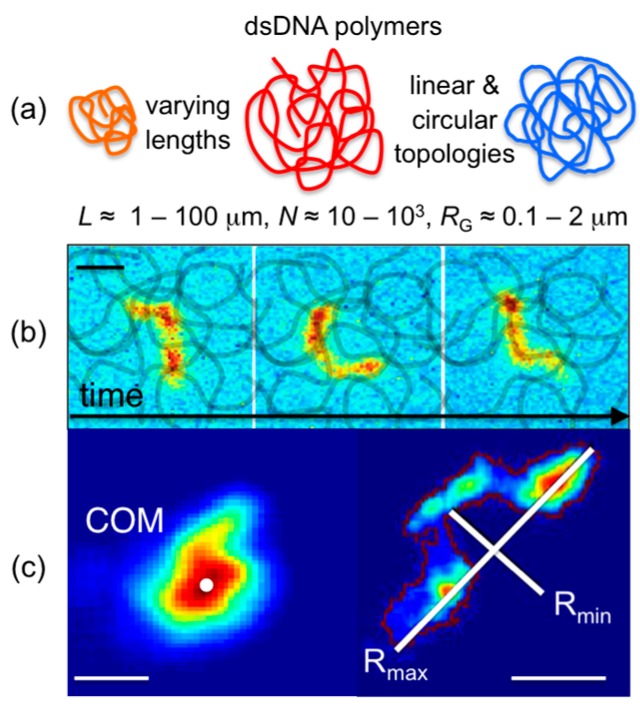
Single-molecule DNA tracking enables steady-state dynamics of entangled polymer molecules to be directly measured. (**a**) DNA molecules of varying sizes and topologies assume random coil conformations with radii of gyration *R*_G_ several times larger than the optical resolution limit, enabling high-fidelity tracking of COM positions as well as molecular conformations. (**b**) Single fluorescent-labeled DNA molecules diffusing among unlabeled entangled DNA molecules are imaged over time using wide-field fluorescence microscopy and high-speed video capture. (**c**) Single-molecule tracking techniques measure the *x* and *y* center-of-mass (COM) positions of each DNA molecule over time, and determine COM mean-squared displacements and corresponding diffusion coefficients *D*. Advances in tracking techniques have also enabled tracking of DNA shape over time, measuring the lengths and orientations of the major and minor axes (*R*_max_ and *R*_min_) from frame-to-frame [33,34,36]. Scale bars in all images represent 1 μm.

A number of intercalating dyes with high DNA-binding affinities, quantum yields that are amplified ~10^3^× upon DNA binding, and spectrally distinct excitation/emission spectra are commercially available (YOYO-1 (491/509), YOYO-3 (612/631), TOTO-1 (514/533), TOTO-3 (642/660); Thermofisher) to enable simple uniform fluorescent-labeling of any DNA construct. High binding affinities allow for imaging of single labeled DNA molecules mixed with high concentrations of unlabeled entangled DNA to track single entangled polymers (Figure 1). Single-molecule imaging and tracking capabilities are further enhanced by the relatively large sizes of DNA, with radii of gyration *R*_G_ ≈ 0.1–2 μm (several times larger than the optical resolution limit). Beyond simple center-of-mass tracking, the conformations of DNA can also be resolved in steady-state conditions (Figure 1) as well as under stress, flow, or confinement [13,76,77].

Further, the persistence length of DNA can be altered by varying the ionic strength of the aqueous solvent, thereby enabling investigations of the role of flexibility on entangled polymer dynamics [10,73]. Finally the ends of DNA can be chemically modified (e.g., biotinylated) to bind to treated microspheres (e.g., streptavidin coated) to enable optical trapping and stretching of single DNA molecules [2,4,6]. Thus, DNA is amenable to a wide range of experimental techniques from single-molecule to macroscopic scales and from steady-state to nonlinear regimes.

The following sections review the application of these techniques to entangled DNA, and the key results and insights each application has contributed to understanding entangled polymer dynamics.

## 3. Single-Molecule Tracking

Single-molecule tracking of fluorescent-labeled DNA molecules embedded in unlabeled DNA has been carried out over the past ~20 years to directly measure diffusion coefficients of entangled DNA. In this technique, wide-field fluorescence microscopy and high-speed video capture (typically using an intensified CCD or CMOS camera) are used to image single diffusing molecules, measure the *x* and *y* center-of-mass (COM) positions of each DNA molecule over time, and determine diffusion coefficients via <*Δx^2^*> = <*Δy^2^*> = *2Dt*.

**Figure 2:**
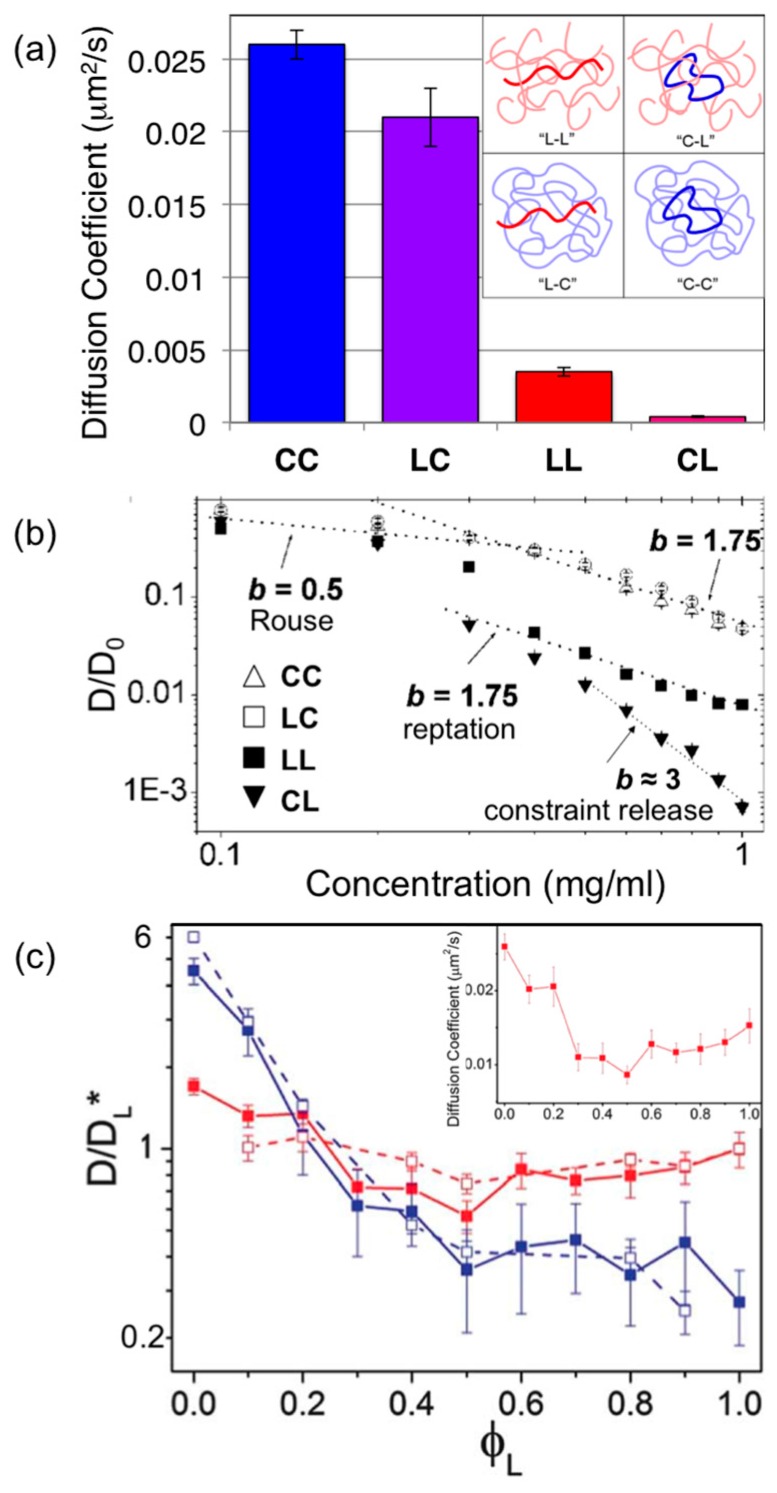
Single-molecule tracking experiments of entangled ring and linear DNA show topology has a dramatic effect on DNA diffusion. (**a**) Measured diffusion coefficients of single linear (L) and relaxed circular or ring (C) 45-kbp DNA in entangled 1.0 mg/mL solutions of 45-kbp linear or ring DNA: ring DNA diffusing among rings (blue, CC), linear DNA diffusing among ring DNA (purple, LC), linear DNA in linear DNA (red, LL), and ring DNA among linear chains (magenta, CL). (**b**) Diffusion coefficients *D* normalized by corresponding dilute values *D*_0_ for concentrations *c* = 0.1–1 mg/mL. Scaling of *D* with concentration shows agreement with Rouse scaling for *c* < 6c* (~0.3 mg/mL) and reptation scaling for *c* > 6c*. The CL case, a ring diffusing among linear chains, shows a stronger dependence on concentration, with a scaling exponent of ~3, demonstrating that constraint release is the dominant diffusive mechanism due to threading events. (**c**) Diffusion coefficients for ring (blue) and linear (red) DNA diffusing in entangled ring-linear blends of varying fraction of linear chains *φ*_L_ = *c*_L_/(*c*_L_ + *c*_R_). Diffusion coefficients are normalized by the corresponding value for a purely linear solution (*D*_L_(*φ*_L_ = 1)). Dashed lines are results of simulations mimicking experimental conditions. Inset is a zoomed-in version of linear DNA diffusion data showing an unexpected non-monotonic dependence of *D*_L_ on blend fraction, resulting from a second order effect of the surrounding constraining ring DNA being threaded by linear DNA and thus nearly immobile. Adapted with permission from references [22], copyright (2007) National Academy of Sciences, USA; [32], published by Royal Society of Chemistry, 2015; [79], published by American Chemical Society, 2007.

This technique was first carried out for lambda DNA to demonstrate that diffusion obeyed reptation scaling laws *D~L*^−2^*c*^−1.75^ [78]. However, in these experiments, only the test chain length *L* was varied (using 48.5, 23.1, 9.4, 6.5 and 4.4 kbp λ DNA fragments) and concentrations were *c* = 0.4–0.8 mg/mL, corresponding to ~10–20× the overlap concentration c*. Similar measurements for linear and ring DNA of lengths 5.9, 11.1, 25 and 45 kbp and concentrations *c* = 0.1–1 mg/mL were carried out to characterize the crossover from semidilute to entangled regimes and determine the role that topology plays in entanglement dynamics (Figure 2) [22,79]. These studies showed that for concentrations above 6c* both entangled ring and linear DNA diffusion exhibited reptation scaling *D*~*L*^−2^*c*^−1.75^. Below this critical entanglement concentration *c*_e_ ≈ 6c*, diffusion followed Rouse scaling *D*~*L*^−1^*c*^−0.5^ predicted for unentangled, semidilute polymer solutions in which hydrodynamic interactions are screened. While in the dilute and semidilute regimes ring DNA diffused only ~1.35× faster than linear chains, entangled rings (1 mg/mL, 45 kbp, ~2*c*_e_) diffused ~10× faster than entangled linear chains. Thus, while reptation appears to dominate diffusion of both ring and linear DNA, the effect of end closure reduces the timescale of reptation confinement, due to more mobile constraints, leading to faster diffusion.

**Figure 3:**
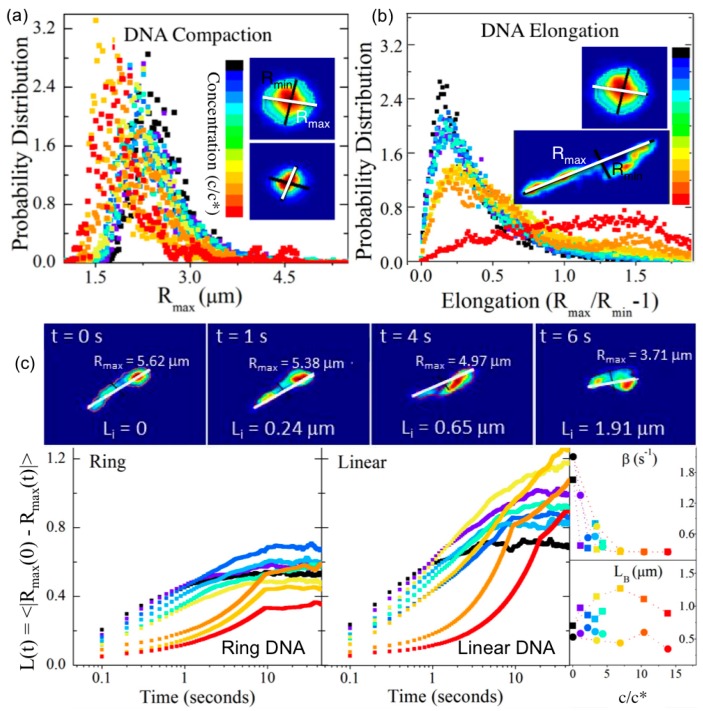
Single-molecule conformational tracking (SMCT) enables time-resolved COM and shape dynamics of entangled DNA to be simultaneously measured. All data shown are for linear and ring 115 kbp DNA in entangled dextran (500 kDa) at concentrations of 0 (black) up to 15c* (red). (**a**) Histograms of the time-dependent major axis lengths (*R*_max_(*t*)) measured in each frame (10 fps) for all measured molecules (~1000) show the probability distribution of DNA sizes for each concentration. Displayed data show that ring DNA compacts by ~20% from dilute random coil conformation as *c* increases. (**b**) Histograms of the elongation or eccentricity factor ((*R*_max_/*R*_min_)–1) determine the corresponding probability distributions of conformational shapes diffusing DNA assume, quantifying the deviation from conformational sphericity. Displayed data show linear DNA elongates ~2× from random coil conformation as *c* increases. (**c**) Measuring the ensemble-averaged frame-to-frame difference in major axis lengths, *L*(*t*) = <(*R*_max_(*t*)–*R*_max_(*0*))>, quantifies the spatiotemporal scales over which DNA fluctuates between varying conformational states. Fitting each concentration-dependent *L*(*t*) curve to an exponential function (*L*_B_–exp(−*β*_B_*t*)) quantifies the rate *β*_B_ at which DNA conformations fluctuate or “breathe” between different states and the corresponding breathing lengthscale *L*_B_. Time-lapse images depict the measurement of the fluctuation length *L_i_* for each frame for a single linear DNA molecule *i*. Displayed data show that while linear and circular DNA fluctuate at equal rates (*β*_B_ ≈ 2.5 s^−1^) at high dextran concentrations, *L*_B_ for linear DNA increases while *L_B_* for circular DNA decreases as *c* increases. Adapted with permission from references [33], published by Biophysical Society, 2015; [34], published by Royal Society of Chemistry, 2015.

Tracer linear DNA diffusing in entangled circular DNA (LC) and tracer rings in entangled linear DNA (CL) were also examined (Figure 2) [22,79]. Linear chains diffusing among circles exhibited nearly identical mobility to circles diffusing in the same solution (*D*_CC_ ≈ 1.3*D*_LC_). However, when both topologies were diffusing among linear chains, ring DNA exhibited markedly slower diffusion; with a concentration scaling exponent close to 3 (compared to 1.75 prediction for reptation), and a diffusion coefficient ~10× slower than that for linear tracers (i.e., *D*_LL_ ≈ 10*D*_CL_) and ~100× slower than ring DNA entangled by rings (i.e., *D*_CC_ ≈ 100*D*_CL_) (Figure 3). This drastic slowing is presumably due to threading of the ring DNA by surrounding linear chains, which prevents reptation. A threaded ring can only diffuse via the threading linear chains releasing their constraints (CR) by reptating out of the center of the ring.

To determine how these marked topology-dependent differences arise, subsequent experiments measured ring and linear DNA diffusion (11, 45 kbp) in blends of ring and linear DNA of varying ratios of 0–100% linear DNA (*φ*_L_ = 0–1) and overall concentrations that span from unentangled to entangled regimes (*c* = 0.1–1 mg/mL) (Figure 2) [32]. Results showed that for the highest concentration as the volume fraction of linear DNA (*φ*_L_) increased to 1 (pure linear DNA solution), *D*_C_ initially rapidly decreased, up to a factor of ~8 at *φ*_L_ = 0.4, then continued to decrease, but with a weaker *φ*_L_ dependence. Interestingly, *D*_L_ displayed a non-monotonic dependence on the linear fraction *φ*_L_, steadily decreasing by a factor of ~3 as *φ*_L_ increased to 0.5, followed by a steady increase, nearly doubling over 0.5 < *φ*_L_ < 1. Corresponding simulation studies showed that this unique non-monotonic dependence of linear chain diffusion on blend fraction is a second order effect of the mobility of the surrounding polymers confining each diffusing polymer. Namely, rings threaded by linear chains are much slower than purely entangled rings or linear chains, thus linear chains diffusing in a *φ*_L_ = 0.5 network (i.e., every surrounding ring is threaded by a linear chain) will be the most rigidly confined and exhibit the most restricted mobility.

Recent advances to single-molecule COM tracking techniques have been developed to measure the conformational dynamics of single diffusing molecules. Single-molecule conformational tracking (SMCT) uses image analysis techniques to measure and track the major and minor axes of fluorescent-labeled linear and ring DNA (Figure 1 and Figure 3) [33,34]. In these studies, time-dependent lengths of the major and minor axes (*R*_max_(*t*), *R*_min_(*t*)), as well as COM positions, of single diffusing DNA molecules are measured to quantify ensemble and temporal distributions of the size (<*R*_max_(*t*)>) and shape (<(*R*_max_(*t*)/*R*_min_(*t*))–1>) of molecular conformations. Measuring the frame-to-frame difference in major axis lengths also enables measurement of the rate *β*_B_ at which DNA conformations fluctuate or “breath” between different states and the corresponding breathing lengthscale *L*_B_. Explicitly, the fluctuation length *L*(*t*) = <(*R*_max_(*t*)–*R*_max_(*0*))> can be fit to an exponential function (*L*_B_−exp(*−β*_B_*t*) to determine *β*_B_ and *L*_B_. SMCT was carried out for ring and linear DNA (11, 115 kbp) embedded in entangled 500-kDa dextran solutions (0%–40% *w*/*v*, 0–15c*), and results showed that for the highest concentrations, linear DNA assumed ~2× elongated conformations, while ring DNA exhibited compact spherical conformations that were ~20% smaller than the random coil configuration volume [33,34]. In contrast, both DNA topologies in dilute phase and when entangled by DNA (at much lower *w*/*v*) assumed spherical random coil configurations with *R*_G_ for linear DNA being ~1.6× larger than that for rings [5].

Cumulative Area (CA) tracking techniques have also been developed to simultaneously measure diffusion modes and rates as well as conformational relaxation times of entangled fluorescent-labeled linear and circular DNA [36]. CA analysis defines regions of five pixels that correspond to the area of each single molecule in each *i*th video frame (corresponding to time point *t_i_*); and for each time point *t_i_* the area for *t_i_* and all of the preceding time points *t_i-1_*, …, *t*_1_ are superimposed to determine the CA. Diffusion coefficients are determined by calculating the mean frame-to-frame differences in CA (MCAD) as a function of lag time *Δt*. Diffusive modes are determined by fitting the mean CA (rather than MCAD) to a power-law function of *Δt*, and comparing scaling exponents to those of simulated 1D random motion, corresponding to reptation and double-folded reptation models (for rings), and 2D random motion, corresponding to either unconfined Brownian motion or lattice-animal motion. Relaxation times are determined by fitting the temporal fluctuations of the CA to an exponential decay with time. Lastly, the distributions of diffusion coefficients (i.e., the standard deviation of distributions) are compared to simulated distributions to reveal molecular individualism and potential unpredicted broadness of single molecule conformational states and dynamics. CA tracking was carried out for linear and ring Charomid DNA in 1 mg/mL solutions of linear or ring DNA solutions. The LL condition followed predictions of the reptation model, as MCAs showed 1D random motion for areas up to 3 μm^2^ (comparable to molecular size), after which data followed 2D random motion (as tube constraints only persist for lengthscales up to the polymer size). CC data fell in between 1D and 2D diffusion (up to 2 μm^2^), potentially reflecting anisotropic conformations of cyclic chains in concentrated solutions. CC diffusion coefficients were also >10× larger than those for LL and CL as opposed to the predicted 4× increase (due to an effective length *L*/2 of extended rings compared to linear chain length *L*). CL data clearly followed a 2D random motion model at all lengthscales with ~3× lower diffusion coefficients compared to LL, likely due to the isotropic motion of a circle threaded by linear chains. CL diffusion also displayed a broader distribution of diffusion rates, suggesting both threading events and entanglements play a role in diffusion. While dilute ring and linear DNA displayed similar relaxation times *(τ*_0_ ≈ 0.12 s), CC conditions relaxed significantly more quickly (~1.25*τ*_0_) than LL (~20*τ*_0_) and CL (~110*τ*_0_) systems, suggesting that entangled ring molecules are far less confined by intermolecular interactions and entanglements. These findings are at odds with the corresponding diffusion data that showed ~10× slower diffusion for concentrated rings compared to the dilute case [22]. Collective results suggest that the surrounding “obstacles”, which are fixed in tube model extensions to ring polymers (i.e., lattice-animal models), are moveable on the timescale of the entangled ring motion, leading to “mutual relaxation” of the constraints and the entangled ring itself (rather than these two mechanisms occurring on well-separated timescales).

Characterizing end-segment motion and reorientation dynamics of entangled DNA over timescales that span from below the entanglement time *τ*_e_ (i.e., the time it takes for the segments to “feel” the tube constraint, or reach a distance *a* from its contour) to above *τ*_D_ has also been accomplished by simultaneously imaging spectrally-distinct DNA end-segments, labeled with quantum dots, and DNA random coils, uniformly labeled with YOYO-1 [80]. The use of quantum dots, with higher signal-to-noise and less photobleaching than conventional dyes, enabled imaging at frame rates up to 90 s^−1^ for up to ~100 s to quantify segmental mean-squared displacements for times <*τ*_e_ (typically only feasible in simulations). To characterize end-segment dynamics, researchers define a frequency-dependent complex diffusivity, analogous to viscoelastic moduli derived from tracking embedded microspheres in passive microrheology experiments (see Section 5). End-to-end squared displacements <*Δx*^2^>, tube radii *a*, number of entanglements per chain *Z*, and relaxation times (*τ*_e_, *τ*_R_ and *τ*_D_) can be extracted from the diffusive moduli. To measure reorientation dynamics, YOYO-labeled DNA is tracked and the time-dependent radius of gyration tensor is calculated. The autocorrelation of the tensor orientation is calculated to extract the reorientation time. Measurements were carried out for linear λ DNA of *c* = 0.2–1.5 mg/mL, and end-segment tracking results showed scaling in accord with predicted scaling of <*Δx^2^*> for all time scales. Namely, <*Δx^2^*>~*t*^1/2^, *t*^1/4^, *t*^1/2^ and *t*^1^ for *t* < *τ*_e_, *τ*_e_ < *t* < *τ*_R_, *τ*_R_ < *t* < *τ*_D_, and *τ*_D_ < *t* respectively. For concentrations exceeding 0.6 mg/mL, data agreed with predicted scaling laws for entangled polyelectrolytes with screened electrostatics: *Z~c*^1.31^, *τ*_e_~*c*^−2.31^, and *τ*_R_~*c*^0.31^. *τ*_e_ ranged from 0.015 s to 0.1 s while Rouse times were all ~0.2 s. The reorientation time quantitatively agreed with *τ*_D_, exhibiting *c*^1.62^ scaling (~2.8–16 s), demonstrating that orientations are lost upon disengagement from the original tube. The end-to-end distance and tube radius, on the other hand, displayed a sharper than predicted decrease with concentration, possibly arising from CR or CLF mechanisms not predicted by classical reptation models.

While the described experiments and methods to study molecular conformations and dynamics have all used DNA, recent comparable studies, using small angle neutron scattering (SANS) and neutron spin echo (NSE) spectroscopy, have been carried out on melts of synthetic polyethylene oxide (PEO) ring polymers [39,40]. These studies have shown that entangled PEO rings exhibit sub-diffusion (<*Δx*^2^>~*t*^<1^) at short time scales but transition to normal diffusion (<*Δx*^2^>~*t*^1^) at longer times. Researchers also demonstrate that PEO rings assume highly compact non-Gaussian conformations, and that CLF and CR play a weaker role than previously assumed.

## 4. Single-Molecule Manipulation

**Figure 4:**
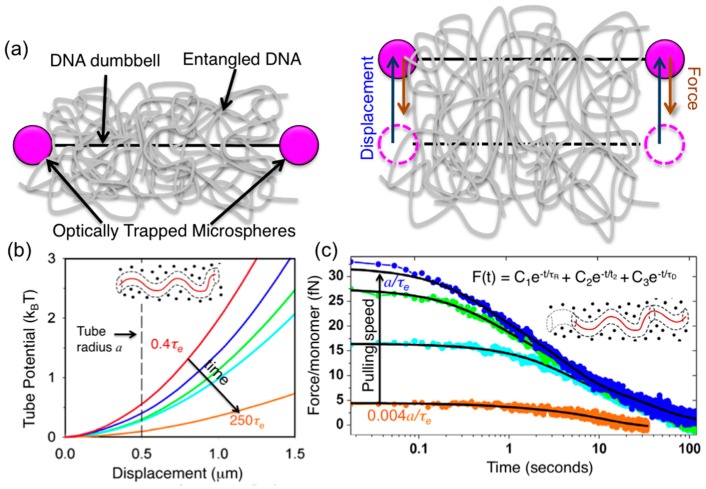
Dual force-measuring optical tweezers are used to characterize the nature of the “tube” field confining a single entangled DNA molecule. (**a**) A 25-kbp DNA-microsphere dumbbell embedded in 115 kbp entangled linear DNA is trapped by dual optical tweezers and pulled perpendicular to its contour at varying speeds while the resulting force imposed on the DNA is measured during and following displacement. Pulling speeds of 0.1 (orange)–65 μm/s (red) were comparable to the predicted equilibrium fluctuation speed *a*/*τ*_e_ ≅ 25 μm/s, corresponding to 0.004*a/τ*_e_–2.6*a/τ*_e_. Varying speeds probed the time dependence of the confining field over timescales 0.4*τ*_e_–250*τ*_e_. (**b**) Integration of measured force during strain showed that the confining field was harmonic; and the measured effective tube radius, defined as the distance at which the confining potential per monomer reached *k*_B_*T*, was *a* ≅ 0.8 μm, compared to the classically predicted *a* ≅ 0.5 μm (dashed line). The confining potential weakened over time, decreasing in response to decreasing pulling speeds, in line with recent non-classical predictions. (**c**) Measured relaxation of force following strains were analyzed via inverse Laplace transform and subsequently fit to a sum of three exponential decays (black lines). The timescales associated with the three decays (*t*_1_ ≅ 0.45 s, *t*_2_ ≅ 5.4 s, *t*_3_ ≅ 34 s) were separated by over an order of magnitude and were independent of the pulling speed, revealing three distinct relaxation mechanisms. *t*_1_ and *t*_3_ were comparable to the Rouse time *τ*_R_ ≅ 0.6 s and the disengagement time *τ*_D_ ≅ 40 s, while the intermediate timescale ~12*τ*_R_ could only be described as recently predicted non-classical “residual stretch relaxation”. Adapted with permission from reference [6], published by American Physical Society, 2007.

Optical tweezers has been used extensively over the past few decades to manipulate single DNA molecules and measure single-molecule properties and dynamics [1,4,9,81]; however, its application to understanding entangled DNA molecules has been more limited in scope. In optical tweezers applications, single DNA molecules are attached at one or both ends to dielectric microspheres (beads), typically via biotin+streptavidin or digoxygenin+antidigoxygenin bonds, and the bead(s) are trapped by the optical tweezers. The bead-attached DNA is then pulled or strained by moving the trap relative to the sample chamber (via piezoelectric control of the trapping beam or the sample chamber) (Figure 4). The seminal application of this approach to entangled systems was that of Steven Chu′s group in 1994, which was the first experimental work to directly demonstrate the existence of tube confinement [2]. In this work, a single fluorescent-labeled linear DNA molecule (*L* ≅ 100 μm), attached to a bead and embedded in a 0.6 mg/mL solution of λ DNA, was dragged and stretched into a series of contorted shapes, and the subsequent relaxation was visualized using fluorescence microscopy. Results showed for the first time that relaxation or recoil occurred primarily along the contour (or within the entanglement tube) of the DNA chain (i.e., reptation).

Subsequent studies by Robertson et al. used dual force-measuring optical tweezers to characterize the nature of the confining “tube” field from the surrounding polymers [6]. In these experiments, a 25-kbp DNA-bead dumbbell, embedded in a 1 mg/mL solution of 115-kbp linear DNA was trapped at both ends and dragged perpendicular to its contour at varying speeds while the resulting force imposed on the DNA was measured during and following displacement (Figure 4). Pulling speeds were comparable to the predicted equilibrium fluctuation speed of the entangled polymer segments, *a*/*τ*_e_, ranging from ~3*a*/*τ*_e_ to ~0.004*a/τ*_e_. Force measurements during strain showed that the confining field was harmonic; and the measured effective tube radius was *a* ≅ 0.8 μm, compared to *a* ≅ 0.5 μm predicted by the DE tube model. However, the confining potential was time-dependent, weakening over time, in line with recent simulations and theoretical extensions to classical tube models [82,83]. Analysis of the force relaxation following the strain revealed three distinct relaxation mechanisms with well-separated timescales (*t*_1_ ≅ 0.45 s, *t*_2_ ≅ 5.4 s, and *t*_3_ ≅ 34 s). *t*_1_ and *t*_3_ were comparable to the classically predicted Rouse time, *τ*_R_ = 6*R*_G_^2^/3π^2^*D* ≅ 0.6 s, and disengagement time, *τ*_D_ = (18*R*_G_^2^/*a*^2^)*τ*_R_ ≅ 40 s, respectively. However, the intermediate timescale ~12*τ*_R_ could only be explained by extensions of the tube model that predict a slower “residual stretch relaxation” mode arising from the tube diameter shrinking during deformation as its length is increased, prohibiting complete elastic relaxation of chain extension to *L* in time *τ*_R_ [6,64]. Reptation is also predicted to occur on this time scale, allowing for weakly entangled systems to configurationally relax on timescales <*τ*_D_.

Similar experiments carried out for entangled ring DNA of the same length and concentration showed that the tube radius *a*_C_ was ~25% smaller than that for linear DNA (*a*_C_ ≅ 0.75*a*_L_ ≅ 0.6 µm), and the confinement field was weaker, displaying a subharmonic dependence on displacement [84]. The measured tube radius was close to predictions based on the pom–pom ring (PPR) model that applies the pom–pom model concept of lattice-tree polymer configurations, originally developed for branched polymers, to entangled rings [49]. The model predicts a tube radius for rings of *a*_C_ = *(N*_eC_*/N*_eL_*)*^1/2^*N*^−1/5^*a*_L_, where *N*_eC_ and *N*_eL_ are the topology-dependent length between entanglements, which equates to *a*_C_ ≅ 0.7*a*_L_ ≅ 0.3 μm for the DNA system studied [85]. Similar to measurements on entangled linear DNA, these experiments also measured three relaxation timescales (*t*_1_ ≅ 0.3 s, *t*_2_ ≅ 4.1 s, *t*_3_ ≅ 11 s) with *t*_1_ close to predictions for Rouse relaxation (*t*_R_ ≅ 0.14 s), *t*_2_ ≈ 13*τ*_R_, and *t*_3_ similar to PPR predictions for the disengagement time *τ*_DC_ = [(*a*_C_^2^/*a*_L_^2^)*N*^−2/5^]*τ*_DL_ ≅ 0.2*τ*_DL_ ≅ 8 s [85].

## 5. Microrheology

**Figure 5:**
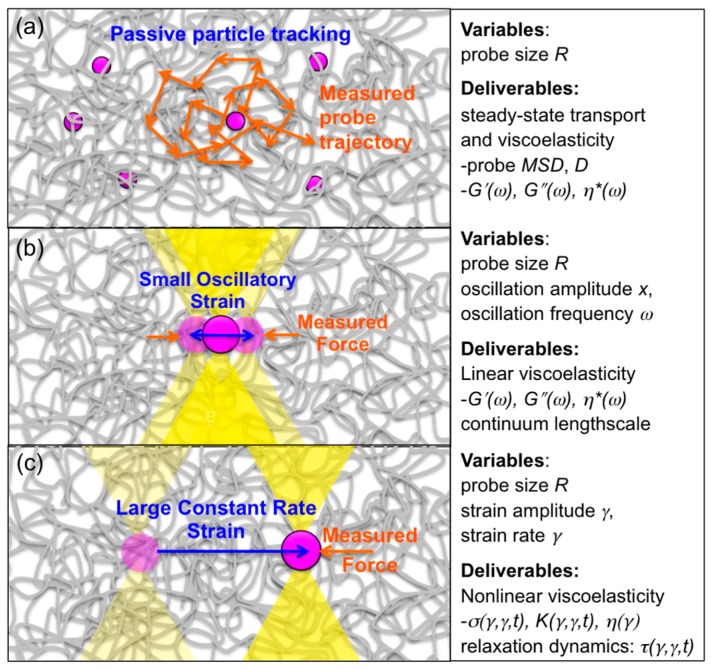
Particle-tracking techniques and force measuring optical tweezers enable microscopic rheological measurements—microrheology—of entangled DNA. (**a**) Passive microrheology tracks the motion of embedded microsphere probes diffusing through entangled DNA, and uses Stokes–Einstein relations to relate probe transport to steady-state viscoelastic properties of the surrounding DNA. Active microrheology can probe linear (**b**) and nonlinear (**c**) viscoelastic properties by measuring the force the entangled DNA exerts on a moving probe to resist strain. The trapped probe is precisely moved relative to the sample by piezoelectric control of the trap or the sample chamber. The resulting force on the probe is measured using a position sensing detector that records the time-resolved laser deflection. (**b**) Small-amplitude (<500 nm) sinusoidal oscillations over a wide range of frequencies (*ω* = 10^−1^–10^3^ rad/s) and with varying probe sizes (*R* = 1–10 μm) enable measurements of linear viscoelastic moduli (*G′*, *G″*, η*) and non-continuum effects. (**c**) Large amplitude (*x* = 1–50 μm) strains of varying rates $\dot{\gamma }$, all faster than the intrinsic relaxation rates of entangled DNA (*Wi* = $\dot{\gamma }$*τ_D_* > 1) probe nonlinear regime viscoelastic properties. Measurements provide nonlinear stress-strain relationships (*σ* vs. *γ*, $\dot{\gamma }$) viscosity *η*, differential modulus *K*, and relaxation mechanisms and timescales *τ*; and can quantify nonlinear phenomena including stress stiffening, yielding, thinning, tube dilation/shrinking and chain stretching.

Microrheology techniques have enabled measurement of the molecular-level and microscale mechanical response of entangled DNA (Figure 5). Typical “passive” microrheology techniques involve tracking passively diffusing microspheres embedded in a complex fluid or soft material and using the microsphere (probe) transport to infer properties of the surrounding fluid. In contrast, “active” microrheology is carried out by actively perturbing the fluid with an optically or magnetically trapped microsphere and measuring the force the fluid exerts to resist the strain. This approach is analogous to bulk rheology techniques, which apply macroscopic strains to a fluid and measure the bulk stress imposed to resist the strain. Both passive measurements and active small-amplitude oscillatory measurements probe the well-behaved linear response of the system in which the viscoelastic characteristics (i.e., *G′*, *G″* and the corresponding complex viscosity η* = (*G′*^2^ + *G″*^2^)^1/2^/*ω*) are independent of strain amplitude and vary with strain frequency.

Linear oscillatory microrheology measurements have been carried out on 1 mg/mL entangled linear DNA of three different lengths (11, 45, 115 kbp), corresponding to 2.4c*, 12c* and 24c* (0.3*c*_e_, 2*c*_e_, and 4*c*_e_) to quantify the frequency-dependent viscoelastic moduli *G′* *G″* and η* [86]. Measurements showed that the 11 kbp system exhibited terminal regime mechanics, *G′~ω^2^* and *G″~ω*, over most of the frequency range (0.5–100 rad/s) as well as a frequency-independent viscosity of ~2 × 10^−3^ Pa s, confirming that very few entanglements are present in the system. Conversely, the 115-kbp solutions displayed expected entanglement mechanics with *G″~ω*^−1/4^, *G′~ω*^0^, and η*~ω^−1^, while the 45-kbp system displayed an intermediate response. Measurements also exhibited a length-independent plateau modulus *G*_N_^0^*~N*^0^ (as predicted by DE theory) of ~0.2 Pa. Motivated by discrepancies between microrheology and bulk rheology measurements, experiments on the same three DNA systems were carried out with probes of radii *R* = 1, 2.25 and 3 μm. Experiments characterized the non-continuum effects that can arise for polymer systems such as DNA with intrinsic lengthscales (i.e., *a*, *R*_G_, and *N*_e_) that are comparable to or larger than the scale of the probe *R*; and determined the probe size necessary to measure continuum mechanics in these systems and match bulk rheological measurements. Researchers showed that for well-entangled systems (115 kbp, 1 mg/mL) measurements are only independent of *R* and reflect bulk measurements (both continuum response characteristics) for *R* > 3*a*, in line with recent predictions of *R* ≈ 5*a* [87]. For marginally entangled systems non-continuum mechanics were displayed as a measured disengagement time, deduced from *ω_c_*, that scaled linearly with probe volume (*τ*_D_~*R*^3^).

An alternative method for using optical tweezers to measure rheological properties of entangled DNA is to extract viscoelastic moduli from the power spectrum *S*(*ω*) of the thermal fluctuations of a loosely trapped bead embedded in entangled DNA. This technique was used to determine the viscoelastic properties (*G′*, *G″*) of calf thymus DNA (~13 kbp) at concentrations *c* = 1–10 mg/mL [88]. For *c* > 2 mg/mL, *G′* was up to two orders of magnitude higher than *G″* (*δ* ≈ 10^−2^), with no apparent crossover frequency *ω*_c_ and a plateau for *ω* > 600 rad/s. Lower frequency *G′* data exhibited power law dependence on frequency with an exponent of ~0.8 for *c* > 0.5 mg/mL.

Passive microrheology measurements, tracking 1 μm probes in 0.2–0.8 mg/mL λ DNA solutions, have also extracted frequency-dependent viscoelastic moduli, low-shear viscosities, and disengagement times for DNA solutions that span the entanglement transition [89]. Reptation dynamics emerged for *c* ≥ 10c* (≥0.6 mg/mL), in which *G′* exceeded *G″* (*δ* < 1) and approached a frequency-independent plateau (*G*_N_^0^) at frequencies of ~0.2–10 s^−1^. The scaling of the number of entanglements per chain, derived from the elastic modulus, agreed with predicted scaling *Z*~*c*^1.31^ for an entangled polyelectrolyte with screened electrostatics. The disengagement time, derived from *ω*_c_, also agreed with predicted scaling *τ*_D_*~c*^1.62^.

Particle tracking microrheology was also used to measure the probe MSD, viscosity, and heterogeneity of supercoiled circular and linear DNA (*L* = 2.9, 5.4, 10.5 kbp, *c* = 0.1–1.6 mg/mL) to assess how DNA topology, concentration, and length affect the distribution of viscoelastic properties [90]. Results showed supercoiled DNA was less viscous than linear DNA, with higher degrees of heterogeneity in the corresponding distributions, but the concentration dependence of the viscosity was stronger than that for linear DNA. Probe trajectories showed that solutions of supercoiled DNA behaved largely like viscous liquids, with undetectable elastic moduli, while the ensemble-averaged elastic modulus for linear 10.5 kbp DNA at 1.6 mg/mL was ~0.5 Pa. For all concentrations of supercoiled DNA, *η* increased monotonically with concentration, while *η* for linear DNA increased slowly for *c* < 0.4 mg/mL after which it rapidly increased. Statistical analysis of the MSD and viscosity distributions showed that supercoiled DNA dynamics were more heterogeneous than linear DNA and the degree of heterogeneity increased with increasing concentration and DNA length. In dilute conditions (*c* < c*) DNA topology did not influence the dynamics, with both linear and circular DNA molecules forming liquid solutions of similar (low) viscosity with minimal heterogeneities.

**Figure 6:**
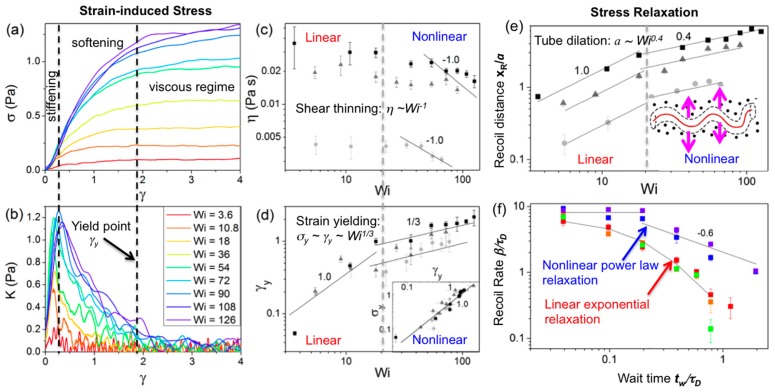
Nonlinear active microrheology of entangled linear 45 kbp DNA reveals nonlinear microscale dynamics for *Wi* > 20. (**a**) Measured stress (*σ* = *F*/*πR^2^*) induced in a 1.0 mg/mL solution of 45-kbp linear DNA, vs. strain (*γ* = *x*/*2R*) for constant strain rates (legend in (**b**)) showing stress-stiffening at short times (~20–200 ms), softening/yielding at intermediate times (0.2–5 s), followed by a terminal viscous regime (all separated by dashed lines). (**b**) Corresponding differential modulus, *K* = *dσ*/*dγ*, vs. *γ* for each *Wi* (*v* = 1–60 μm/s) further demonstrating nonlinear stiffening (increasing *K*) and yielding (decreasing *K*) phenomena. (**c**) Apparent viscosity *η* vs. *Wi* for 0.3 (circles), 0.5 (triangles), and 1.0 mg/mL (squares) solutions show nonlinear shear thinning scaling *η*~*Wi*^−1^ for *Wi* > 20 (dashed line). (**d**) Yield strain *γ*_y_ vs. *Wi* for DNA solutions in (**c**) with predicted scalings for linear (*γ*_y_~*Wi*^1.0^) and nonlinear regimes (*γ*_y_*~σ*_y_*~Wi*^1/3^). (**e**) Maximum recoil (relaxation) distance *x_R_* reached by the probe (upon immediate release after strain) vs. *Wi*, shows agreement with predictions for linear regime scaling (*x*_R_~*Wi*) for *Wi* < 20, crossing over to scaling in accord with predicted nonlinear tube dilation (*a*~*Wi*^0.4^) for *Wi* > 20. (**f**) Recoil rate, *β* vs. wait time *t*_w_ for *Wi* = 3.6 (red), 10.8 (orange), 18 (green), 54 (blue), and 108 (violet) displays classical linear behavior, i.e., single exponential relaxation (black line), for *Wi* < 20, while *Wi* > 20 data show agreement with recent predictions for two-phase power law relaxation, *τ*_D_^−1^~*t*^0^, *t*^−0.6^ (black lines), in the nonlinear regime, demonstrating simultaneous reptation and contraction/healing of dilated tubes. Adapted with permission from reference [91], published by American Physical Society, 2015.

Nonlinear (large strain) constant rate microrheology measurements have also been carried out for 45 kbp linear DNA at concentrations *c* = 0.3, 0.5 and 1 mg/mL using a large strain distance (*x* = 30 μm) and a range of strain speeds (*v* = 1–60 μm/s) to characterize the crossover regime from linear to nonlinear response and determine the strain-rate dependence of the nonlinear response for entangled DNA (Figure 5 and Figure 6) [91]. The speeds corresponded to Weissenberg numbers of *Wi* = $\dot{\gamma }$*τ_D_* = 3.6–126 (where $\dot{\gamma }=3v/\sqrt{2}R$) [92] and the force *F* exerted by the entangled DNA on the probe was measured during the strain (*γ* = *x*/*2R*) to determine the stress (σ = *F*/π*R*^2^) versus strain curve, the differential modulus *K* = *d*σ/*dγ*, the yield stress and strain (σ_y_, *γ*_y_) and the steady-state viscosity. At a precise wait time *t*_w_ after the strain, the probe was released from the optical trap and its recoil trajectory was measured to characterize the confinement and relaxation dynamics. Measurements demonstrated a distinct crossover to nonlinear mechanics at *Wi* ≈ 20. For *Wi* > 20 the force response displayed stress-stiffening (increasing *K*) at short times (~20–200 ms), yielding/softening (decreasing *K*) at intermediate times (~0.5–2 s), and shear-thinning *(η*~*ω*^−1^). The *Wi* > 20 response also exhibited power-law stress relaxation and features that signified tube dilation [59,65,67,69,71,72,76,93]. While nonlinear entanglement reduction or tube dilation has been suggested to be due primarily to chain stretching [67,93,94], chain stretch has been shown to result in stress overshoots [76,93], in which the σ vs. *γ* curve reaches a maximum before decreasing to a steady-state plateau value. While stress overshoots have been seen in macrorheology studies on entangled DNA (described below), the described microrheology measurements show no such overshoot, suggesting that apparent stretching is a macroscale response that only arises from collective stretching of many chains [76,94]. Further, measurements were in agreement with a recently proposed model, originally developed for rigid rod polymers, that does not account for stretching [70,72]. The measured yielding dynamics were also in accord with this model, with σ_y_*~γ*_y_ and *γ*_y_*~Wi*^1/3^ scaling arising from strain-induced dilation of the entanglement tube (reduction in entanglement density). Probe recoil trajectories for varying wait times *t*_w_ = 0–20 s following strain, which sense the relaxation of induced DNA deformations, were fit to single exponentials to calculate recoil rates *β* and maximum recoil distances *x*_R_. For *Wi* < 20, recoil rates displayed the expected exponential dependence on *t*_w_, in accord with classical DE predictions and macroscopic rheology results [76,93]. However, for *Wi* > 20, recoil rates exhibited a two-phase power-law dependence (*β~t*_w_^0^ for *t*_w_ < 0.2 s, *β~t*_w_^−0.6^ for *t*_w_ > 0.2 s), in agreement with the tube dilation model of Ref [72], suggesting nonlinear flow-induced tube dilation near the local strain is not apparent at the macroscale. Immediately following the strain (*t*_w_ = 0) the maximum recoil distance for *Wi* < 20 was comparable to the tube radius *a* and scaled with *Wi*, as expected for linear regime dynamics; however, for *Wi* > 20, *x*_R_ reached up to ~3*a* and scaled as *Wi*^0.4^, in agreement with the predicted rate dependence of tube dilation [70,72].

Magnetic tweezers have also been used to access the nonlinear regime of entangled λ DNA (*c* = 1.4 mg/mL) [94]. In contrast to the constant strain rate nonlinear optical tweezers measurements described above, magnetic tweezers measurements apply constant force strains (by applying a constant force to a trapped probe) and measure the resulting probe trajectory (rather than induced force). The creep response curves resulting from applied forces *F* = 1–1000 pN, measured over 0.2–15 s, displayed Stokes response for *F* < 2 pN where steady state velocity is quickly reached and scales linearly with *F*. For larger forces, this initial Stokes response is followed by a nonlinear shear thinning response, in which the probe accelerates, and then reaches a higher terminal steady state speed. Corresponding constant rate bulk rheology measurements measured the evolution of shear stress as a function of time. While bulk viscosity of λ DNA showed a linear response at $\dot{\gamma }$ = 0.1 s^−1^ (monotonically increasing with time), viscosity overshoots and shear thinning became apparent at $\dot{\gamma }$ = 30 s^−1^ ≈ 100*τ*_D_^−1^ (*Wi* ≈ 100) indicating nonlinear response. Comparison of data to simulations based on the ROLIE-POLY model (ROuse-CCR tube model for LInear Entangled POLYmers) for entangled polymers [66], suggest that the nonlinear response is a result of both significant chain stretching/retraction and entanglement rearrangements. While these nonlinear relaxation mechanisms result in shear thinning at the microscale, the signature nonlinear feature at the macroscale is stretch-driven overshoots.

## 6. Microfluidics

**Figure 7:**
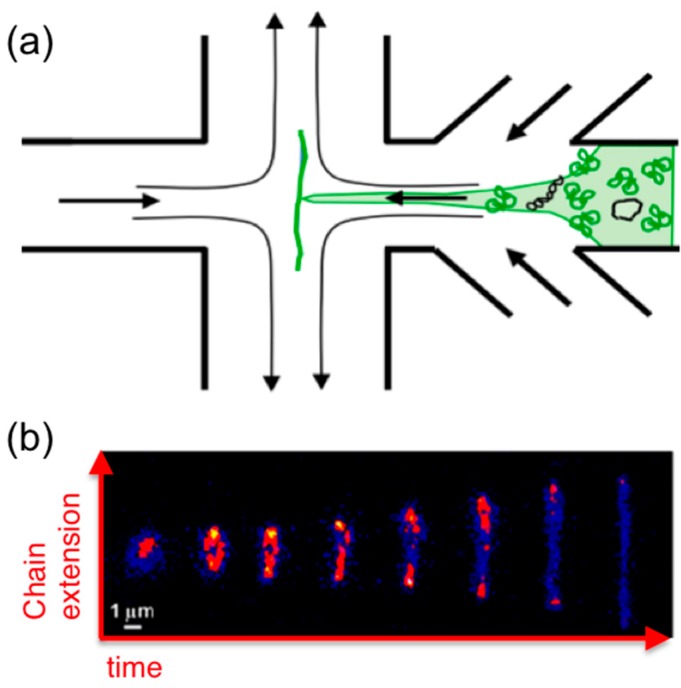
Microfluidic devices enable visualization of DNA extension and relaxation during extensional strain. A microscale extensional flow is directly imposed on a DNA sample (**a**) and a single fluorescent-labeled molecule is imaged via fluorescence microscopy (**b**) during the flow to characterize conformational deformation. (**a**) Extensional flow is created with a “microfluidic trap” that applies a planar *x–y* extensional flow to the sample by controlled flow of fluid (and sample) into the chamber from −*x* and +*x* channels which exits the chamber through +*y* and −*y* channels thereby trapping a fluorescent-labeled molecule in the stagnation point in the center of the chamber. (**b**) Molecular conformations of single trapped molecules are imaged while applying repeated extensional flow and cessation to characterize flow-induced molecular extension and relaxation. Sample extensional relaxation of dilute 45-kbp ring DNA during flow is shown. Adapted with permission from references [13], published by Royal Society of Chemistry, 2012; [35], published by American Chemistry Society, 2015.

Microfluidics offer an alternative approach to measuring molecular-level response to strain. While the majority of these studies focus on single-molecule and dilute dynamics, microfluidics have been used to probe semi-dilute and entangled DNA systems. While in microrheology embedded probes perturb or deform the system and the resistive force exerted by the polymers on the probe is measured, in typical microfluidics experiments, a microscale flow is directly imposed on the sample to deform the polymers and a sparse amount of fluorescent-labeled molecules are imaged via fluorescence microscopy during the flow to characterize conformational deformation (Figure 7).

Controlled shear flows can be created using microscope chambers outfitted with one (or 2) motor-controlled surface(s) (e.g., the top microscope slide) that can be precisely moved, in a direction parallel to the surface (*x, y*), while keeping the other surface (e.g., the bottom coverslip) fixed (or moving it in the opposite direction). This geometry allows for feedback-controlled shear, perpendicular to the optical axis (*z*), to be applied to the sample during imaging. This configuration has been used for λ DNA at *c* = 10, 16, 23 and 35c* to measure: (i) the relaxation following fast shear; (ii) the response during steady shear flows (1.3–5.4 s^−1^); and (iii) the transient response during sudden constant shear flow (1.3 s^−1^) [76]. (i) The relaxation of fractional extension of DNA exhibited an exponential decay with time with 2 distinct timescales comparable to those reported in Ref [6]. While no timescale corresponding to *τ*_R_ was found, a timescale ~10*τ*_R_ was measured, similar to the intermediate “residual stretch” relaxation timescale reported in Ref [6] arising from tube stretching/shrinking and corresponding enhanced friction that slows the elastic relaxation. Slow relaxation timescales were also comparable to predicted *τ*_D_ values (~10–92 s for 10c*–35c*), and scaled as *t*_slow_*~c^3.3^*, in agreement with reported separability timescales of bulk nonlinear shear relaxation moduli, and suggesting that both CR and CLF contribute to relaxation; (ii) Fractional extension trajectories during constant shear flow showed broad distributions, not predicted by DE theory, with fractional variances of up to 50% even at shear rates below *τ*_R_*^−1^* (the nominal linear regime); (iii) Transient extensional responses to shear flow showed an equally broad distribution of trajectories with some molecules undergoing extreme cyclic stretching and contraction while others maintained steady modest extension during the entire strain. The collective data exhibited qualitative features of the ROLIE-POLY model but lacked quantitative agreement, possibly arising from the broad molecular individualism not accounted for in the theory. Subsequent primitive sliplink chain simulations were carried out to attempt to resolve these discrepancies and capture the reported single-molecule measurements [95]. These simulations showed improved agreement with the distribution of molecular extensions and the response to startup shear, but were still unable to quantitatively capture dynamics. Authors suggest that these discrepancies arise from differences between global conformational dynamics and segmental intermolecular dynamics, including the lack of sensitivity to CCR exhibited by conformational extension data. Alternative sources of discrepancies could be the effect of DNA charge or shrinking/dilation of confinement tubes, none of which are accounted for in simulations.

Extensional flows can also be created with “microfluidic traps” that apply planar *x*–*y* extensional flows to samples by controlled flow of fluid into the chamber from −*x* and +*x* channels which exits the chamber through +*y* and −*y* channels, thereby trapping a molecule in the stagnation point in the center of the chamber (Figure 7) [13]. Molecular conformations of single trapped molecules are imaged while applying repeated extensional flow and cessation to characterize flow-induced molecular extension and relaxation. These techniques were recently used to characterize the extensional dynamics of single ring DNA molecules (25, 45, 115 kbp) subject to flows of *Wi* ≈ 0.1–3 in the highly dilute regime [35]. The extensional relaxation time of ring DNA scaled with molecular length as *L*^1.41^, versus *L*^1.71^ for linear DNA (48.5, 288, 340 kbp), likely due to the reduced importance of excluded volume for smaller ring DNA. The transition from coil to stretch for rings also occurred at ~1.25× higher *Wi* compared to linear chains with a correspondingly smaller distribution of stretching pathways (i.e., less molecular individualism). However, these studies have yet to be extended to concentrated solutions, so how topology impacts the extensional dynamics of entangled DNA remains unknown.

## 7. Macrorheology

While the majority of entangled DNA studies have been single-molecule or microscale experiments, bulk rheology or “macrorheology” has also been carried out for entangled linear DNA. Both linear and nonlinear rheology measurements of λ DNA at *c* = 10, 16, 23 and 35c* were carried out for oscillation frequencies *ω* = 10^−2^–10 s^−1^ and shear rates $\dot{\gamma }$ = 10^−2^–10^4^ s^−1^ [76]. The two highest concentrations displayed scaling of viscoelastic moduli representative of entangled polymers with CCR and CLF contributions; namely, *G′* approached a frequency-independent plateau (*G*_N_^0^ ≈ 2.7 Pa), *G″~ω*^−1/4^ and *η*~*ω*^−1^. The crossover frequencies for the three highest concentrations yielded a scaling of *τ*_D_*~c*^0.43^. The shear stress σ displayed a rate-independent plateau at intermediate rates, likely arising from CCR alleviating stress build up, followed by a high frequency σ~$\dot{\gamma }$ regime, suggested to arise from significant chain stretching and potential CCR-induced kinks. Transient viscosity following sudden inception of shear flow showed stress-overshoots for $\dot{\gamma }$ > 1 s^−1^, supporting the importance of chain stretching to the macroscopic response. However, the overshoot time decreased for increasing shear rates (*τ*_peak_~$\dot{\gamma }$^−0.9^) rather than remaining constant as theoretically predicted. The nonlinear relaxation modulus was also measured to show that relaxation for all concentrations displayed two distinct relaxation modes, qualitatively similar to those reported in Ref [6]—an initial fast relaxation (~0.1 s), due to chain retraction, followed by slow reptation.

Linear oscillatory measurements were also carried out on linear calf thymus DNA (~13 kbp) of *c* = 1–10 mg/mL (~3–30c*) [96,97]. The measured viscoelastic moduli (*G′*(*ω*), *G″*(*ω*)) displayed concentration-dependent scaling of the crossover frequency and plateau modulus, *ω*_c_*~c*^−2.4^ and *G*_N_^0^*~c^2^*^.3^, in line with predictions for entangled semiflexible polymers with appreciable excluded volume [96]. The data for the lowest concentrations were, however, in closer agreement with semi-dilute models, as expected for *c* < 6c* [97].

No previous bulk rheology experiments have been carried out on entangled ring DNA but there have been a limited number of recent rheology studies using synthetic ring polymers synthesized via LLLC methods for producing highly pure rings [42,45]. Linear rheology measurements of melts of both synthetic rings and linear polymers of varying molecular weights show that the ratio of zero-shear viscosities for entangled rings versus linear chains scales with molecular weight as *η*_L_/*η*_R_*~N*^1.2^ [42]. This scaling is weaker than previous predictions (*N*^1.6^) and simulations (*N*^2^) [26,98], attributed to minute linear contaminants and ring–ring concatenation. Subsequent linear and nonlinear macrorheology measurements on marginally entangled ring melts demonstrate that in the linear regime ring dynamics are better described by the Rouse model rather than lattice-animal models [45]. In the nonlinear regime rings also exhibited less shear thinning and a much weaker stress overshoot than similar linear polymer melts [45].

## 8. New Combined Techniques

Several advances aimed at bridging single-molecule techniques with macrorheology measurements have recently been developed.

Standard rheometers have been integrated with confocal fluorescence microscopes to enable bulk shearing of entangled DNA solutions while simultaneously imaging single fluorescent-labeled DNA molecules or microspheres spiked into the solution. This method, which enables coupling of bulk stress measurements to time-resolved molecular conformations and velocities, has been applied to entangled solutions of calf thymus DNA (~75 kbp, *c* = 5, 10 mg/mL) to investigate the molecular origin of wall slip and shear banding that can arise in complex fluids and soft materials [77,99,100]. To investigate wall slip, entangled DNA solutions (with a sparse amount of YOYO-labeled DNA) were placed in a chamber that was surface-treated to encourage strong DNA–surface interaction [77]. DNA velocity *V*, measured by tracking the displacement of stained DNA as a function of time for varying heights *Y* in the chamber was used to determine the apparent shear rate *dV/dY* = $\dot{\gamma }$_app_, where *Wi* = $\dot{\gamma }$_app_*τ*_D_. For *Wi* < 1, stress growth was monotonic in time (implying no-slip elastic deformation), the normalized stress scaled as σ*/G*_N_^0^*~Wi*, and DNA molecules throughout the chamber remained coiled over the entire strain. However, for *Wi* > 1, σ/*G*_N_^0^ exhibited a wide *Wi*-independent stress plateau, and while stress growth was initially monotonic, it quickly reached a stress overshoot followed by a plateau. During *Wi* > 1 strain, DNA at the surface initially began as coiled and entangled, yet after the stress overshoot, adsorbed DNA disentangled from the bulk surrounding DNA chains (slip), causing its speed to increase from 0 to *V_S_* with *V*_S_~*Wi*. After the slip transition DNA also elongated in the direction of shearing indicating that chain stretching likely contributes to slip. In the slip regime, the applied bulk shear rate scaled as *Wi*^0.25^, rather than *Wi* (for no slip conditions), and slip length scaled as *Wi*^0.75^. Significant conformational changes were also evident in imaged molecules for *Wi* > 70, as occasionally molecules stretched by the shear would recoil and tumble, evidence of full disengagement from the entanglement network.

Similar methods were also used to investigate shear banding—inhomogeneous network deformation with bands of high and low strain—in entangled calf thymus DNA (*c* = 5, 11, 22 mg/mL) spiked with silver-coated microspheres [99,100]. When subjected to a sudden startup shear (*Wi* = 780) a stress overshoot was evident with shear banding occurring after the stress maximum, suggested to arise from a localized collapse of the network. For increasing applied shear rates $\dot{\gamma }$, the velocity profiles following overshoot (*V* vs. *Y*) remained nearly identical, indicating the onset of stress yielding. While strong shear banding resulted from conventional startup shear, slow rate ramp up and quench down produced homogeneous strain throughout the steady-state stress plateau. Further, for the most entangled networks, as the applied stress hit the overshoot, the velocity profile displayed a localized negative velocity, indicative of a cohesive breakdown and recoil, that ended as the applied stress dropped to a minimum (preceding the steady-state plateau regime). Nearly permanent shear banding was observed in the terminal plateau regime, with homogeneous shear only reemerging at very long times (~100τ_D_). This reemergence was coupled to wall slip, which was further shown to be able to prevent entangled DNA from breaking up into shear bands.

Bulk extensional viscosity (*η*_e_) measurements have been carried out on dilute (1/2c*) and semidilute (c*) solutions of λ and T4 DNA by measuring the filament thinning and drop breakup of the bulk fluid thread [101]. By carrying out measurements in a flow focusing microfluidic device, researchers were able to simultaneously measure the bulk extensional viscosity (as a function of strain rate) and image single DNA molecules in the fluid thread. The presence of DNA significantly increased the filament thread length and the breakup time. Semidilute T4 DNA displayed viscosity thinning and a fluid filament thinning profile that exhibited two exponential decays, indicating that the elastic stresses from the DNA can act as stabilizers even without entanglements. During thinning single fluorescent-labeled molecules underwent a coil-stretch transition with a broad distribution of coil-stretch times and fractional extension lengths. However, no such measurements have been carried out for entangled DNA or ring DNA.

## 9. Conclusions and Outlook

DNA offers a powerful experimental platform for elucidating dynamics of entangled polymer solutions and is uniquely suited for: (i) investigating the role of polymer topology and concentration; and (ii) spanning orders of magnitude in measurement lengthscales (microscopic to macroscopic) and timescales (linear to nonlinear regimes). The experiments conducted over the past two decades have directly validated key theoretical concepts and predictions of the acclaimed reptation tube model, but have also shed light on the molecular dynamics responsible for discrepancies between traditional tube theory and experimental results, establishing new theoretical extensions and alternatives as accurately modeling dynamics.

Steady state dynamics are now well understood and characterized for monodisperse linear DNA and blends of linear and ring DNA. However, rheological studies, using microscale and macroscale methods are needed to systematically probe the concentration and length dependence of DNA response in the entangled regime. Most of the rheological studies to date have been on only a few different concentrations and lengths, so they have been unable to test established scaling laws relating intrinsic polymer properties to viscoelastic moduli. The results of future comprehensive studies that systematically vary length, concentration and topology will not only test classical scaling laws, but could potentially reveal alternative scalings, motivating new theoretical studies to resolve the revealed conflicts between established theory and experiment.

Measurements have also elucidated the controversial confinement properties associated with entangled ring DNA and ring-linear blends, and the applicability of tube model concepts to entangled rings. However, all of the circular DNA measurements have been molecular-level steady-state measurements, so how robust the confinement models are to applied stress or strain remains to be determined. Different response and relaxation dynamics exhibited by entangled linear DNA at varying lengthscales suggests that while ring DNA may relax more quickly at the microscale, many chains entangled together may lead to more pronounced entanglement effects at the macroscopic scale. Further, how threading contributes to the response to shear remains untested.

Finally, with the wide range of techniques applicable to entangled DNA, experiments have been able to delineate between the importance of various non-classical phenomena at different lengthscales and timescales. While CCR and CLF appear to be important across scales; tube dilation, shear thinning, yielding, banding and slip only emerge at nonlinear strain rates. Further, stretching appears to only appreciably contribute to macroscopic response, while molecular individualism and tube dilation only play a principal role at the microscale. However, measurements that can bridge the gap between molecular-level and macroscopic dynamics to probe the elusive mesoscale are necessary to determine the lengthscale at which each relaxation or response mechanism emerges and to characterize the lengthscale-dependence of its contribution as measurements move from molecular-level to macroscale. The lengthscale beyond which collective many-polymer interactions, rather than individual entangled polymer dynamics, dominate network response and how stress propagates from the molecular-level to the mesoscale to produce such distinct responses at varying lengthscales remain important unanswered questions. Techniques that build on the combined techniques described—combining recent steady-state image analysis advances with rheology-microscopy instrumentation—will enable visualization and characterization of the molecular deformations and network rearrangements that give rise to the apparent scale-dependent features.

## Figures and Tables

**Table 1:** Properties of available DNA constructs used in entangled polymer studies. The listed constructs are commercially available (* see text for vendors) or available upon request from the Robertson-Anderson lab. The degree of polymerization *N* is determined using a Kuhn length of *L*_K_ = *2L*_p_ = 100 nm. Topology-dependent radii of gyration *R*_G,L/C_ are determined from measured dilute diffusion coefficients [5]. Overlap concentrations c* = (*M*/*N*_A_)/(4/3π*R*_G_^3^) and entanglement concentrations *c*_e_ ≈ 6c* listed are calculated from measured *R*_G_ values for linear DNA (*R*_G,L_). Italicized numbers are approximates based on the average length of polydisperse calf thymus.

DNA construct	Length *L* (kbp)	Length *L* (μm)	*N* (*L/L*_k_)	*R*_G,L_ (μm)	*R*_G,C_ (μm)	c* (mg/mL)	*c*_e_ (mg/mL)
pYES	5.9	1.97	19.7	0.22	0.13	0.152	0.914
pPIC11	11.1	3.70	37.0	0.28	0.17	0.127	0.762
calf thymus 1 *	*13*	*4.33*	*43.3*	*0.31*	*0.20*	*0.116*	*0.695*
pCC1FOS-25	25	8.33	83.3	0.45	0.26	0.071	0.427
Charomid *	42.2	14.1	141	0.61	0.40	0.048	0.291
pCC1FOS-45	45	15.0	150	0.63	0.41	0.046	0.277
lambda (λ) *	48.5	16.2	162	0.66	0.43	0.044	0.263
calf thymus 2 *	*75*	*25.0*	*250*	*0.85*	*0.55*	*0.032*	*0.190*
CTD-2342K16	115	38.3	383	1.06	0.65	0.025	0.149
T4 *	168	56.0	560	1.35	0.88	0.017	0.103
CTD-2657L24	289	96.3	963	1.98	1.26	0.010	0.057
